# Application of Chan-Lam cross coupling for the synthesis of N-heterocyclic carbene precursors bearing strong electron donating or withdrawing groups

**DOI:** 10.1038/srep12431

**Published:** 2015-07-23

**Authors:** Liliang Huang, Chengxiang He, Zhihua Sun

**Affiliations:** 1 College of Chemistry and Chemical Engineering, Shanghai University of Engineering Science, 333 Longteng Road, Shanghai 201620, China

## Abstract

Chan-Lam cross coupling allowed efficient synthesis of N,N’-disubstituted *ortho*-phenylene diamines bearing strong electron donating or withdrawing groups, such as nitro or methoxy groups, with moderate to high yields. These diamines can then be turned into N-heterocyclic carbene precursors after condensation with trimethyl orthoformate. The same strategy can also be utilized for the synthesis of N-monosubstituted aniline derivatives containing a functionalized *ortho*-aminomethyl group as intermediates for chiral 6-membered ring carbene precursors.

As one of the fastest developing classes of catalyst in organic synthesis, N-heterocyclic carbenes (NHCs) are widely used in aldol condensation^1^,^2^,^3^,^4^,^5^, Stetter reaction^6^,^7^,^8^,^9^, Diels-Alder reaction^10^, olefin metathesis^11^,^12^ and a variety of carbon-heteroatom cross coupling reactions^13^,^14^,^15^,^16^,^17^. Commonly used NHCs include an imidazole, imidazoline, benzimidazole, or triazole core for the carbene moiety (Fig. 1)^18^,^19^,^20^. Our group has recently reported several benzimidazole-based NHCs for catalysis in organic transformations^21^,^22^. These benzimidazole-based NHCs were traditionally synthesized in a two-step procedure (Fig. 2) mostly using Buchwald-Hartwig cross coupling reaction to construct the key N,N’-disubstituted *ortho*-phenylene diamines^23^ before ring closure with an orthoformate^24^. Although Buchwald-Hartwig reaction has the advantage of using readily available *ortho*-phenylene diamine and organic halides as starting materials, it is well known from previous studies of the Buchwald group that the reaction conditions (high temperature and use of strong base in conjunction with palladium catalyst) often are not compatible with substrates bearing strong electron effecting groups on aromatic rings such as nitro, nitrile, or alkoxy substituted phenyl halides^25^. In our hands particularly, reaction of *ortho*-phenylene diamine with neither nitro nor methoxy substituted phenyl bromide could produce the desired products in sufficient yields (Fig. 2). As our studies venture into the understanding of the effects of various substituents on NHC catalysis, alternative synthetic strategies for the desired NHCs are needed. We sought to investigate if Chan-Lam reaction can be used to replace Buchwald-Hartwig coupling to obtain N,N’-disubstituted phenylene diamines because the mild reaction conditions of Chan-Lam reaction should in theory tolerate a wide range of functional groups^26^,^27^,^28^,^29^,^30^,^31^.

## Results and Discussion

We first chose to optimize reaction conditions for the Chan-Lam cross coupling of phenylene diamine **1** and two separate boronic acids **2a** (bearing *ortho*-OMe) and **2c** (bearing *meta*-nitro) to afford the desired diamine intermediates **3a** and **3c** (Table 1). The investigation covered variations in catalyst loading ratio, amount or identity of base, and reaction time, etc. As summarized in Table 1, higher catalyst loading led to shorter reaction time to completely consume the starting materials but not necessarily higher yields (entries **1**–**6** for synthesizing **3a**, and entries **7**–**12** for **3c**). Triethylamine as the base for the reaction seemed to be adequate as replacement by pyridine or using a mixture of triethylamine and pyridine did not lead to obvious improvements. Based on these results, we chose the condition in entry **3** of Table 1 for coupling with substrates bearing strong electron donating groups, and the condition in entry **7** of Table 1 for substrate containing strong electron withdrawing groups.

Then, we expanded the reaction to larger scope of substrates to prepare the N, N-disubstituted phenylene diamine intermediates, followed by ring closure to form the final carbene precursors. The results are summarized in Table 2 and discussed below.

The 13 entries in Table 2 cover examples of various electron withdrawing or donating groups (NO_2_, F, CF_3_, or OMe) either on the phenylene diamine **1** or on the boronic acid **2**. These all gave satisfactory results for the synthesis of **3** using the optimized conditions found in Table 1, and yields of 51–84% were obtained. Further ring closures with trimethyl orthoformate to form the carbene precursors **4** were mostly smooth and with high isolated yields of 88–95%. The exceptions are the ring closure reactions for **3g** and **3h** bearing a nitro group on the phenylene diamine ring and either a CF_3_ or a F at the *ortho* position on the N and N’ phenyl rings. Probably the strong electron withdrawing groups *ortho* to the aniline moiety makes the diamine extremely unreactive, because no product were detected for ring closures of **3g** or **3h** even when the reactions were carried out at much elevated temperature of 80 °C.

With the above success, we sought to further expand the application of Chan-Lam cross coupling reaction to the preparation of intermediates of another class of carbene precursors. We recently reported a new class of NHCs in which the carbene bearing central cores contain a chiral center and fused aromatic ring^32^. The original synthesis of the precursors utilized Buchwald-Hartwig reaction to functionalize the two key carbene stabilizing nitrogen atoms. As expected, there are two drawbacks for this original synthetic route: 1) low compatibility to strong electron donating or withdrawing groups; and 2) simultaneous modification of both the aromatic and the aliphatic amines leading to loss of control on differentiating substitutions on the nitrogen atoms of the NHC core. Utilizing the Chan-Lam reaction to firstly derivatize the aromatic amino group could potentially overcome both shortcomings. Our results with various substrates are summarized Table 3. In all the six examples, the optimal conditions found for phenylene diamine in Table 1 were no longer useful because that led to slow reactions. We therefore further increased catalyst loading and changed reaction time. For reactions involving boronic acids **2a**, **2g**, or **2h** (entries **1**, **3**, **4** and **5** in Table 3), we increased Cu(OAc)_2_^·^H_2_O to 1.0 eq of **1e** and **1f**, and the reaction time was decreased to around 12 h. For reactions of substrate **2c**, we increased catalyst loading to 0.5 eq of **1** and decreased reaction time to **3h** (entries **2** and **6** in Table 3). These led to yields of 43–76% in the six examples of **3**. All reaction products of the Chan-Lam coupling were mono-functionalized derivatives on the aniline nitrogen atom. This opened up region-control opportunities of the intermediates. To demonstrate this, compounds **3n** and **3q** were selected to be further derivatized using Buchwald-Hartwig reaction to modify the chiral alkyl amine and afforded **3n1** and **3q1** (Table 3), respectively. These were then condensed with trimethyl orthoformate to formed the desired carbene precursors **4n** and **4q** (Table 3) in excellent yields.

## Summary

In summary, we used Chan-Lam cross coupling reaction to successfully incorporate aromatic rings containing strong electron donating or withdrawing groups on phenylene diamines or aminomethyl anilines for the synthesis of NHC precursors. This protocol is a good complementary to the traditional Buchwald-Hartwig coupling reactions. In addition, application of the Chan-Lam reaction can properly distinguish derivatization on aromatic amines over alphatic amines, and therefore provides regio-control for the installation of different substitutions on a class of chiral 6-membered ring carbenes.

## Methods

### General procedure A for synthesis of 3a-s

A mixture of diamine **1** (1.0 mmol), phenylboronic acid **2** (2.1 mmol) was dissolved in dichloromethane (20–30 mL) in a 100 mL round-bottomed flask equipped with a stir bar. Then, Et_3_N (1.0–2.0 equiv) and Cu(OAc)_2_^·^H_2_O (0.2–0.5 equiv) were added at room temperature. The reaction mixture was stirred for 2–48 h. After completion of the reaction (checked by TLC), the mixture was filtered through Celite and washed with EtOAc. The crude product was purified by silica gel chromatography (hexane : EtOAc = 40:1, 20:1 or 5:1) to give the desired product **3**.

### General procedure B for synthesis of N-heterocyclic Carbene precursors 4a-s

Compound **3a-s** (0.5 mmol) was dissolved in trimethyl orthoformate (5 mL). Then, concentrated hydrochloric acid (0.1 mL) was added. The mixture was reacted at room temperature for 1–12 h. Then most solvent was evaporated under reduced pressure. The crude product was purified by silica gel chromatography (dichloromethane: methanol = 10:1) to give the desired product **4a-s**.

## Additional Information

**How to cite this article**: Huang, L. *et al.* Application of Chan-Lam cross coupling for the synthesis of N-heterocyclic carbene precursors bearing strong electron donating or withdrawing groups. *Sci. Rep.* **5**, 12431; doi: 10.1038/srep12431 (2015).

## Figures and Tables

**Figure 1:**
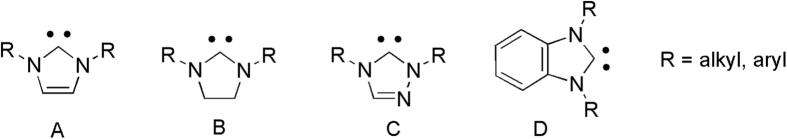


**Figure 2:**
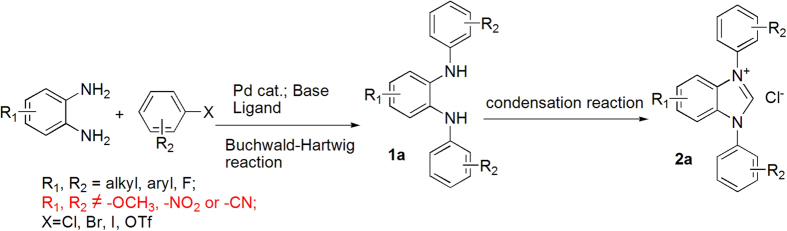


**Table 1:**
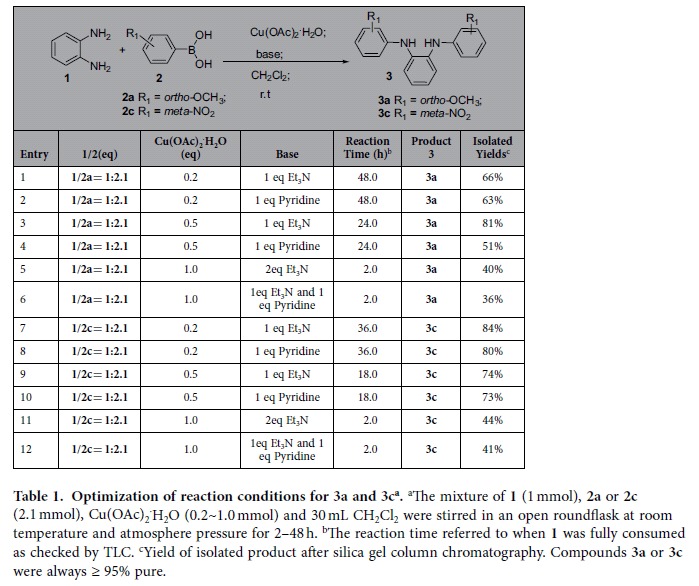
Optimization of reaction conditions for 3a and 3c^a^.

^a^The mixture of **1** (1 mmol), **2a** or **2c** (2.1 mmol), Cu(OAc)_2_^·^H_2_O (0.2~1.0 mmol) and 30 mL CH_2_Cl_2_ were stirred in an open roundflask at room temperature and atmosphere pressure for 2–48 h.

^b^The reaction time referred to when **1** was fully consumed as checked by TLC.

^c^Yield of isolated product after silica gel column chromatography. Compounds **3a** or **3c** were always ≥ 95% pure.

**Table 2:**
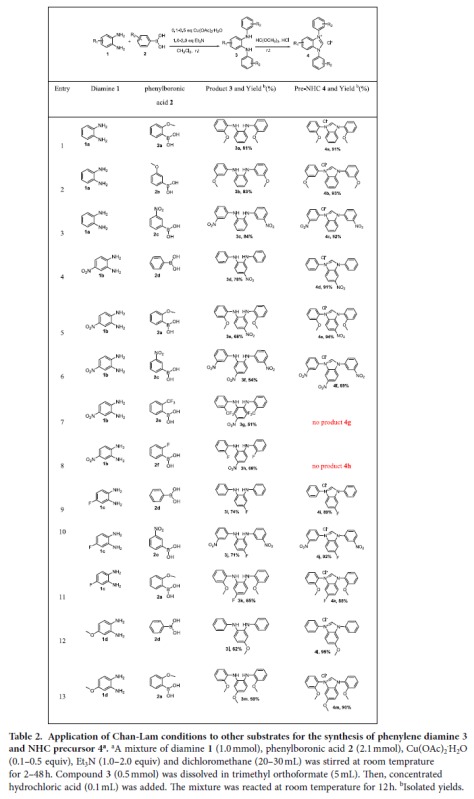
Application of Chan-Lam conditions to other substrates for the synthesis of phenylene diamine 3 and NHC precursor 4^a^.

^a^A mixture of diamine **1** (1.0 mmol), phenylboronic acid **2** (2.1 mmol), Cu(OAc)_2_^·^H_2_O (0.1–0.5 equiv), Et_3_N (1.0–2.0 equiv) and dichloromethane (20–30 mL) was stirred at room temprature for 2–48 h. Compound **3** (0.5 mmol) was dissolved in trimethyl orthoformate (5 mL). Then, concentrated hydrochloric acid (0.1 mL) was added. The mixture was reacted at room temperature for 12 h.

^b^Isolated yields.

**Table 3:**
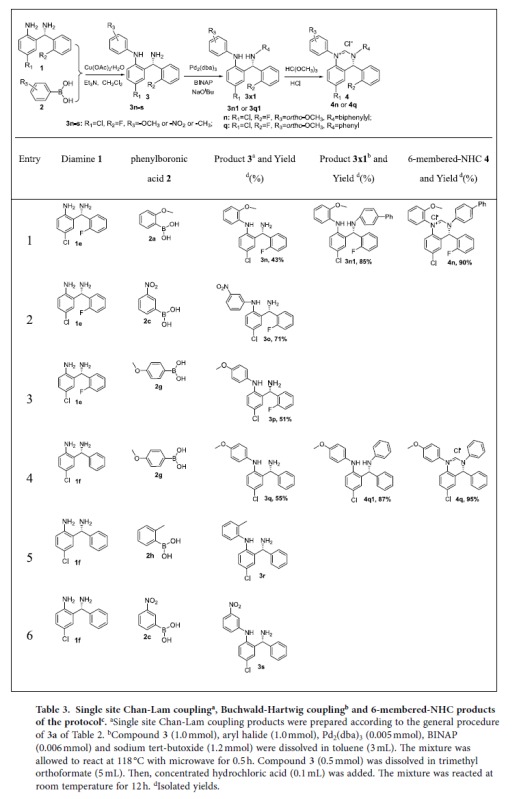
Single site Chan-Lam coupling^a^, Buchwald-Hartwig coupling^b^ and 6-membered-NHC products of the protocol^c^.

^a^Single site Chan-Lam coupling products were prepared according to the general procedure of **3a** of Table 2.

^b^Compound **3** (1.0 mmol), aryl halide (1.0 mmol), Pd_2_(dba)_3_ (0.005 mmol), BINAP (0.006 mmol) and sodium tert-butoxide (1.2 mmol) were dissolved in toluene (3 mL). The mixture was allowed to react at 118 °C with microwave for 0.5 h. Compound **3** (0.5 mmol) was dissolved in trimethyl orthoformate (5 mL). Then, concentrated hydrochloric acid (0.1 mL) was added. The mixture was reacted at room temperature for 12 h.

^d^Isolated yields.
